# Development of the Citrus Longhorned Beetle *Anoplophora chinensis* (Cerambycidae: Coleoptera) on Artificial Diet and Chilling Effect on Their Life Cycle Completion

**DOI:** 10.3390/insects17030285

**Published:** 2026-03-05

**Authors:** Hai Nam Nguyen, Ki-Jeong Hong

**Affiliations:** 1 Department of Agricultural Life Science, Sunchon National University, Suncheon 57922, Republic of Korea; hainguyen309@gmail.com; 2 Plant Protection Research Institute, Hanoi 1910, Vietnam

**Keywords:** artificial diet, chilling duration, chilling temperature, longhorned beetle, pupation

## Abstract

*Anoplophora chinensis* (Coleoptera: Cerambycidae) is an invasive, economically important, quarantined wood-boring pest whose long-life cycle complicates laboratory rearing and management. This study investigated the combined effects of artificial diet, chilling duration, and temperature on pupation cues. Adults collected from the wild were allowed to oviposit, and newly hatched larvae were reared on a prepared artificial diet. Larval weight was recorded biweekly to assess growth and mortality. At 12 weeks of age, larvae were subjected to cold treatments at 5 °C or 10 °C for 9, 12, 14, 16, or 19 weeks, then returned to warm rearing conditions to monitor pupation. Additional chilling cycles were applied when necessary. Pupation percents increased with chilling duration, reaching 55% after 16 weeks at 10 °C compared with 16.7% after 12 weeks and none after 9 weeks. Developmental durations were 34.43, 55.93, and 88.65 weeks for larvae experiencing one, two, and three chilling cycles, respectively. Adults body weight was consistently lower than that of field-collected individuals for both males and females. These findings confirm that chilling is essential for pupation cues and demonstrate that both duration and temperature strongly influence pupation success. Importantly, the combination of artificial diet with optimized chilling regimes enhances pupation rates, providing a practical foundation for mass-rearing protocols of *A. chinensis* to support future research and management programs.

## Simple Summary

This study examined how cold treatment (chilling) affects the development of *Anoplophora chinensis* larvae reared on an artificial diet and their life cycle completion. Larvae were exposed to low temperatures (5 °C and 10 °C) for various durations and then returned to warm conditions to monitor pupation. Results showed that longer chilling periods increased pupation success: 55% pupated after 16 weeks at 10 °C, compared with 16.7% after 12 weeks and none after 9 weeks. Some larvae required one to three chilling cycles to complete their life cycle, with development times ranging from 34 to 89 weeks. The body weight of adults emerging from the artificial diet was significantly lower than those collected in the field. Overall, the study demonstrates that both chilling duration and temperature are critical for successful pupation, offering insights that may support scalable rearing of this species under laboratory conditions.

## 1. Introduction

The citrus longhorned beetle—CLB (*Anoplophora chinensis* Forster, 1771; Coleoptera: Cerambycidae)—is currently recognized as one of the most serious invasive wood-boring pests in Europe and Asia. *A. malasiaca* is regarded as a synonym of *A. chinensis* and was frequently cited in earlier Japanese literature [1]. Morphological variation and the presence of multiple COI haplotypes within the species reflect considerable genetic diversity among populations from different geographic regions [1,2,3]. This polyphagous xylophagous beetle is native to East Asia, particularly China, Japan, and Korea, as well as several Southeast Asian countries, including Vietnam, Malaysia, Indonesia, and the Philippines [1,4]. Following initial incursions into Europe in the early 2000s, primarily linked to imported bonsai and nursery stock [2,4,5], *A. chinensis* remains recorded in several European countries as of late 2025, including Italy, France, Croatia, Germany, the Netherlands, Poland, Slovenia, and Slovakia. In Switzerland, the species has been declared eradicated, while scattered reports also exist from Austria, Belgium, Denmark, Estonia, Finland, Lithuania, and Sweden [5,6]. Species distribution models and climate variables consistently indicate that the CLB has the potential to expand its global range well beyond its native distribution. Europe and North America are identified as regions at particularly high risk. Climate change is projected to increase the extent of suitable habitats, especially at higher latitudes [7,8].

CLB attacks a wide range of broadleaf hosts, including citrus, maple, willow, poplar, and chestnut, causing severe damage through larval tunneling in woody tissues [4,9]. Its life cycle includes oviposition beneath host tree bark, prolonged larval tunneling within woody tissues, pupation in the trunk or root system, and adult emergence during the summer months. This species is considered highly destructive, as larvae bore deeply into the xylem and cambial layers, thereby weakening structural integrity and often resulting in host tree mortality [4].

Early Japanese studies established that the CLB has a multiyear life cycle lasting up to three years, with larval survival strongly influenced by temperature regimes [10]. A previous study further demonstrated the feasibility of rearing larvae on cellulose-based artificial diets, although growth was slower compared to natural hosts [11]. Subsequent work confirmed citrus, ornamental, and forest trees as preferred hosts, with adult females exhibiting higher fecundity and survival [12,13]. Building on these foundations, more recent studies in Europe and China have refined artificial diet formulations and climate control protocols. Favaro et al. (2017) developed a general diet suitable for multiple invasive cerambycids, showing that CLB can complete development but with reduced efficiency relative to *A. glabripennis* Motschulsky, 1854 [14]. Cai et al. (2016) demonstrated that combining artificial diets with controlled temperature regimes (20–30 °C) enables successful rearing from egg to adult [15], while other works highlighted the critical role of temperature, with development arrested at ≤10 °C or ≥35 °C [16,17].

Collectively, these studies underscore both the feasibility and the challenges of mass rearing the CLB, emphasizing that reliable colonies require specialized diets and strict environmental regulation [14,15,17]. Larval development in *A. chinensis* is strongly influenced by exposure to low temperatures [17]. Overwintering larvae can extend their life cycle under fluctuating thermal regimes, with chilling periods helping to synchronize development with seasonal cycles [10,11]. Larvae enter developmental arrest at ≤10 °C and resume growth when temperatures rise, indicating a facultative diapause-like response [10,15]. Chilling is critical for CLB larvae [17]; without this period, development is disrupted, survival rates decline, and adult emergence becomes irregular. Although artificial diets can sustain continuous development under optimal conditions (20–30 °C), the absence of chilling often reduces fitness and longevity [10,15].

Despite evidence that exposure to low temperatures (≈10 °C for ~12 weeks) is critical for *A. chinensis* larvae to complete their life cycle [14,17], several uncertainties remain. Previous conclusions were based on limited experimental designs and did not assess whether shorter or longer durations could also be effective. Moreover, the precise temperature thresholds remain uncertain. To address this gap, the present study expands the range of chilling durations and temperature gradients, aiming to refine our understanding of diapause termination and improve rearing protocols.

In this study, we evaluated the development of *A. chinensis* under artificial diet conditions and examined the effects of chilling duration and temperature on the completion of their life cycle. Our objective was to address the above-mentioned gap in order to refine artificial rearing protocols and strengthen pest management strategies for this destructive longhorn beetle.

## 2. Materials and Methods

### 2.1. Insect Sources

CLB adults were manually collected from willow trees, *Salix alba* L. (Malpighiales: Salicaceae), growing along the Dongcheon River in Suncheon-si, Jeollanam-do, Republic of Korea (34.982874 N, 127.489675 E), with a total of 26 females and 27 males during June and July of 2023 and 2024 (Table 1).

Adults collected from the wild were transferred to the breeding room and weighed for further comparisons. The insects were paired in plastic vessels (10 × 15 × 20 cm) or mass-reared in breeding cages (40 × 40 × 40 cm) at a density of approximately 4–5 pairs to obtain eggs. Water was provided in a 5 oz cup containing soaked cotton on top. Fresh willow twigs served as food, and larger twigs (1–2 cm in diameter; 15 cm long) were placed for oviposition. Food and oviposition substrate were replaced daily. The insects were maintained in the breeding room at a temperature of 24–26 °C under a photoperiod of 16L:8D, with RH ranging from 40–60%.

**Figure 1:**
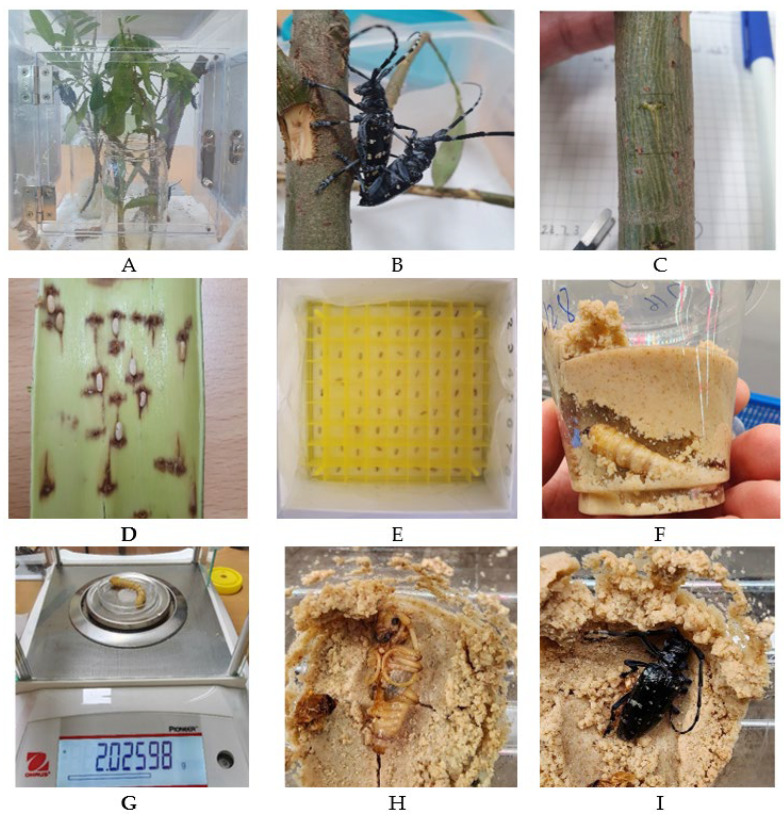
Rearing *Anoplophora chinensis* under laboratory conditions. (**A**) Breeding cage for adults; (**B**) Adults pairing; (**C**) Eggs were deposited under the bark; (**D**) Egg exposed after peeling the bark; (**E**) a Combi Cryo-Box for egg separation; (**F**) Larval feeding on an artificial diet cup; (**G**) Larval weighing; (**H**) Pupation on diet; (**I**) Adult emergence.

The eggs were harvested by gently detaching them from beneath the host plant bark after peeling the twigs. Subsequently, they were surface-sterilized with 5% sodium hypochlorite solution, rinsed with distilled water, and individually placed into a Combi Cryo-Box (SCB4500, 81-hole type; Ecocell, Gyeonggi, Republic of Korea) or a Corning Falcon tissue culture dish (flat bottom 96 wells type, Sigma-Aldrich, St. Louis, MO, USA) to prevent larval cannibalism after hatching (Figure 1).

### 2.2. Artificial Diet Preparation

The diet was modified from the AG2 diet described by Keena (2005) [18] (Table 2). Agar powder was mixed with distilled water and heated to boiling. Wheat germ, sucrose, sodium benzoate, and citric acid were added at boiling, while the remaining ingredients were incorporated after cooling for 5 min. All components were then blended for 3 min. The diet was poured into 1 oz or 5 oz plastic cups, and lids were closed once the diet had cooled to prevent dehydration. The prepared diet was stored in a refrigerator until use.

**Table 2:** Ingredients used in the artificial diet.

Ingredient	Supplier	Unit	Volume	Remark
Distilled water	IS Chemical, Busan, Republic of Korea	mL	900	Solvent
Agar powder	Chemicals Duksan, Incheon, Republic of Korea	g	30	Gelling agent
Wheat germ	Bob’s Red Mill, Milwaukie, OR, USA	g	65	Proteins, lipids
Sucrose	Sigma-Aldrich, St. Louis, MO, USA	g	18	Carbohydrates
Casein from bovine	Sigma-Aldrich, St. Louis, MO, USA	g	9.4	Proteins, amino acids
Yeast extract	Sigma-Aldrich, St. Louis, MO, USA	g	25	Proteins, amino acids
Cellulose powder 38 μm	Fujifilm Wako, Osaka, Japan	g	121	Fiber
Wesson salt	Thermo Fisher Scientific, Waltham, MA, USA	g	0.9	Mineral mix
Vitamin mix *	NOW Foods, Bloomingdale, IL, USA	capsule	4	Vitamins
Sodium benzoate	Sigma-Aldrich, St. Louis, MO, USA	g	2	Preservative
Citric acid	Sigma-Aldrich, St. Louis, MO, USA	g	2	Preservative
Sorbic acid	Sigma-Aldrich, St. Louis, MO, USA	g	1.6	Preservative
Tetracycline	Sigma-Aldrich, St. Louis, MO, USA	g	0.2	Antibiotic

* See Appendix A for detailed vitamin ingredients.

### 2.3. Development of A. chinensis Larvae on the Artificial Diet

Neonate larvae, upon hatching, were transferred into 1 oz artificial diet cups via a small perforation created on the diet surface to facilitate larval entry, and each cup was labelled. Diet cups were replaced at two-week intervals or earlier if signs of fungal or bacterial contamination were observed. Prior to transfer into fresh diet cups, larvae were surface-sterilized by immersion in a 5% sodium hypochlorite solution for 5 s, followed by rinsing in distilled water for an additional 5 s. At six weeks of age, the larvae were relocated to larger 5 oz diet cups to accommodate growth.

In 2023, a total of 84 newly hatched larvae were used to examine the development of *C. chinensis* on an artificial diet. Larval weights were recorded at two-week intervals. All larvae were reared under identical conditions for 12 weeks before being placed in an incubator (VS-1203P1-150-0, Vision Scientific, Daejeon, Republic of Korea) set at 10 °C under dark conditions to stimulate pupation (RH: 20–40%). Following the chilling period, larvae were returned to the rearing room (T = 26 ± 2 °C; RH = 40–60%; 16L:8D photoperiod) and maintained for an additional 12 weeks. Larvae that pupated during this interval were recorded, whereas those that did not pupate were subjected to a second 12-week chilling treatment at 10 °C. After the second chilling, larvae were again reared for 12 weeks under the same room conditions; individuals that still failed to pupate were then exposed to a third chilling treatment, during which the temperature was set at two levels (5 °C and 10 °C) for 16 weeks. The dates of pupation and adult emergence were recorded, and adult body weight was measured 7 days post-emergence to allow comparison with wild-collected adults.

### 2.4. Determining the Effect of Chilling Duration and Chilling Temperature on A. chinensis

The larvae used in this experiment were reared from eggs laid by adults collected in 2024. The diet and rearing methods followed those described above. Because the number of larvae of the same age was insufficient, chilling treatments could not be performed simultaneously. Instead, larvae that had reached 12 weeks of age and attained a body weight of at least 1 g were sequentially placed into cold chambers (VS-1203P1-150-0, Vision Scientific, Daejeon, Republic of Korea) set at 10 °C, with 15–20 individuals per cohort. An additional treatment at 5 °C was conducted with 20 individuals for a duration of 16 weeks. The remaining larvae (17 individuals) were maintained in the rearing room (as above) without exposure to cold treatment.

After accumulating cold exposure durations for 9, 12, 14, 16, and 19 weeks at the two temperature levels, age-matched cohorts were transferred back to the rearing room (T = 26 ± 2 °C; RH = 40–60%; 16L:8D photoperiod) for further observation. The new diet cup was replaced immediately after larvae were reintroduced to the rearing room and subsequently every 2 weeks (as above protocol). The number of pupae obtained under each experimental condition was recorded until larvae had been maintained in the rearing room for an additional 12 weeks, in order to calculate pupation percentage.

### 2.5. Data Analysis

Adult body weight was compared using one-way ANOVA after confirming normality with the Shapiro–Wilk test. Pearson’s chi-square test was used to assess the association between pupation rate and duration of cold treatment. When significant differences were identified, Fisher’s Exact Test was applied for each pairwise comparison, and the resulting *p*-values were compared with the adjusted significance level (α = 0.05) following Benjamini and Hochberg’s method [19]. Data analysis was performed using Minitab v22 (Minitab, LLC, State College, PA, USA) and Excel (Microsoft, Redmond, WA, USA).

## 3. Results

**Figure 2:**
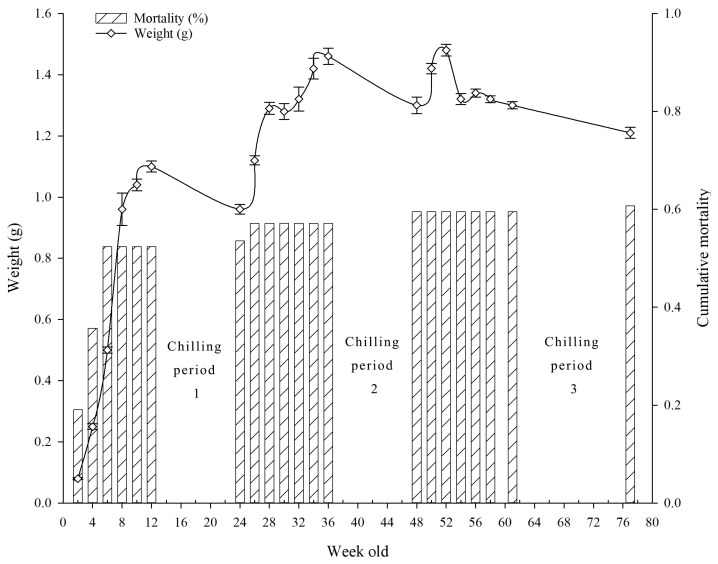
Body weight (line with error bar shows SE value) and cumulative mortality (bar chart) of citrus long-horned beetle larvae *Anoplophora chinensis* reared on artificial diet with three chilling periods (initial number of larvae *n* = 84).

*A. chinensis* was able to develop on an artificial diet. Larval mortality increased sharply during the first 6 weeks (>50%) and then stabilized. After 12 weeks, the mean larval weight reached approximately 1.10 g, rising to 1.46 g after 36 weeks. Certain individuals required up to three cold treatments before completing the immature stages (Figure 2).

The pupation percentage after the first and second chilling was only 7.7–12.9%, whereas in the third treatment, 60% of larvae developed into adults. Development from larval hatching to adult emergence in CLB was completed after one, two, or three chilling periods, corresponding to 34.43, 55.93, and 88.65 weeks, respectively (Table 3).

The body weight of CLB adults reared on an artificial diet was significantly lower than that of wild-collected adults for both females (*p* = 0.001) and males (*p* = 0.036). On the artificial diet, females reached a mean weight of 1.06 g, compared with 1.28 g for individuals collected from the wild. For males, mean weights were 0.67 g on the artificial diet and 0.79 g in the wild (Table 4).

The duration of cold treatment did not affect CLB mortality (*χ*^2^ = 2.785, *df* = 5, *p* = 0.73), but it had a statistically significant effect on pupation percentage after chilling (*χ*^2^ = 22.699, *df* = 5, *p* < 0.01). The percent of larvae pupated under the 16-week chilling treatment was 55%, which was significantly higher than the shorter chilling treatments of 12 weeks, where pupation reached only 16.7%. No individuals pupated under the 9-week treatment. Interestingly, one individual pupated in the non-chilled cohort but failed to develop into an adult (Table 5).

Chilling temperature also had a marked effect on the pupation percentage of CLB. In both the 2023 and 2024 experiments, no individuals pupated following exposure to 5 °C. In contrast, at 10 °C, 55% of chilled CLB larvae pupated in the 2023 experiment and 60% in the 2024 experiment, with subsequent adult emergence. These differences were statistically significant (*p* = 0.002 in the 2023 experiment and *p* < 0.001 in the 2024 experiment) (Table 6).

## 4. Discussion

The present study demonstrates that *A. chinensis* can complete development on artificial diets, albeit with notable constraints. Larval mortality was high during the first six weeks (>50%) before stabilizing, and growth remained slow, with mean larval weights reaching 1.10 g at 12 weeks (prior to the first chilling). These results are consistent with earlier work on *A. chinensis* [14,15] and *A. malasiaca* [11,20], which showed that artificial diets can sustain development but with lower performance compared to natural hosts, and with elevated mortality, particularly among younger instars. In contrast, recent studies emphasized the critical role of temperature in artificial rearing success, demonstrating that fluctuating temperature regimes substantially improved larval survival relative to constant conditions [15,17]. Keena and Richards (2022) further noted that the ecological origin of *A. chinensis* populations may influence survivorship, suggesting that genetic or geographic variation could modulate rearing outcomes [17].

The significantly lower adult weights observed in our study (females: 1.06 g vs. 1.28 g in the wild; males: 0.67 g vs. 0.79 g) reinforce the conclusion that current artificial diets compromise physiological performance, echoing previous reports of reduced fitness and fecundity under laboratory conditions [14]. Cai (2016) similarly found female weights ranging from 0.715–1.464 g and male weights from 0.500–1.129 g across diets and generations, with sawdust-based diets generally producing heavier females [15]. Favaro et al. (2017) likewise documented reduced adult mass and fecundity in laboratory-reared cohorts compared with natural hosts, underscoring that diet composition strongly influences developmental outcomes [14]. Importantly, previous studies revealed that larvae of certain cerambycid species may perform better on drier diets [17,21], a pattern consistent with the outcomes of sawdust-based diets reported by Cai (2016) [15].

Our study did not include direct nutritional analysis, but previous work on cerambycids and other wood-boring beetles has highlighted the importance of protein content, carbohydrate balance, and moisture levels in supporting larval growth and adult fitness [18,22]. This indicates that both nutrient composition and diet texture play key roles. The current diet appears more suitable for later instars, as survival rates were relatively high thereafter. However, comprehensive studies are still needed to determine the optimal nutrient composition and rearing conditions for *A. chinensis*, particularly during the early instars.

For many insect species, diapause and cold exposure are dialectically interconnected, a relationship that is particularly evident in species from temperate and cold regions [23,24,25,26]. These insects enter diapause to enhance their survival during harsh winter conditions, yet they also require the cold period itself to break dormancy and resume development in the warmer spring [23,27]. Empirical evidence demonstrates that numerous species are unable to complete their life cycle without experiencing winter chilling [23,27,28,29]. Understanding the nature of this relationship provides valuable insights into species distribution, informs the development of control strategies, and facilitates the successful rearing of these insects under laboratory conditions.

Cold treatment emerged as a critical factor influencing pupation success in *A. chinensis*. While mortality was unaffected by cold duration, pupation percentage increased significantly with longer chilling periods. Pupation was 55% after 16 weeks of chilling, compared with only 16.7% after 12 weeks, and no pupation after 9 weeks. These results align with Adachi (1994) [10] and Keena and Richards (2022) [17], who emphasized the necessity of chilling for pupation cues, but extended their findings by demonstrating that multiple chilling cycles may be required. In contrast to its sister, *A. glabripennis* generally requires only a single winter chilling period to complete its life cycle [14,18]. Indeed, pupation percentages after the first and second chilling were low (7.7–12.9%), whereas the third treatment yielded 60% adult emergence. This variability highlights heterogeneity in diapause intensity among individuals, a phenomenon also noted in *A. malasiaca*, where overwintering strategies are flexible, and development can extend across multiple seasons [10]. While diapause has been reported in *A. chinensis* [10], we did not measure physiological or endocrine markers to verify this developmental arrest. Larvae in this study were subjected to chilling after reaching 12 weeks of age, a time point chosen to standardize physiological development as suggested previously for *A. glabripennis* [14,18]. However, it remains uncertain whether 12 weeks is sufficient for diapause stabilization in *A. chinensis.* Longer pre-chilling durations (e.g., 14–20 weeks) may allow greater accumulation of energy reserves and endocrine adjustments, potentially enhancing the effectiveness of chilling in terminating diapause of *A. chinensis*. Future studies should therefore examine the optimal larval age at the onset of chilling to refine rearing protocols.

Temperature thresholds further shaped pupation outcomes. No individuals pupated at 5 °C, whereas 55–60% pupated at 10 °C, with subsequent adult emergence. These results clarify that diapause termination requires moderate chilling rather than extreme cold, consistent with Cai et al. (2016) [15], who showed that development arrests at ≤10 °C but resumes under controlled regimes of 20–30 °C. Together, these findings suggest that although chilling is essential, its effectiveness depends on both duration and temperature, with 10 °C representing a functional threshold for pupation cues in *A. chinensis.* A limitation of the present study is that only two chilling temperatures (5 °C and 10 °C) were tested. Consequently, the precise threshold for diapause termination could not be determined, and intermediate temperatures (e.g., 7–9 °C) may also be effective. Subsequent work may benefit from testing a broader set of chilling conditions to refine the temperature requirements for pupation cues in *A. chinensis*.

Cold exposure clearly plays a critical role in the development of *A. chinensis*, and longer periods of chilling may be favored by the species. Our results suggest that extended chilling (e.g., 16 weeks) promotes pupation, yet the underlying mechanism remains uncertain. It is unclear whether prolonged cold directly facilitates successful pupation or whether secondary effects, such as diet desiccation, contribute to improved outcomes [17,21]. Nevertheless, diapause in *A. chinensis* appears to be facultative, allowing some individuals to complete development without experiencing a cold winter [10,15]. In this study, we observed one larva that successfully pupated without chilling, similar to the findings of Cai et al. (2016) [15] and Adachi (1994) [10]. This flexibility helps explain why *A. chinensis* is primarily adapted to temperate and subtropical climates [1,4,6], yet shows a tendency to expand into higher latitudes under climate change scenarios [7,8], where longer winters may be required. At the same time, the species persists in warmer regions of southeastern Asia [4], highlighting its broad ecological tolerance and invasive potential.

Notably, low-temperature treatment of *A. chinensis* resulted in a strongly female-biased sex ratio among emerged adults after the first and second chilling treatments. Although our sample size was limited, similar patterns have been reported in other cerambycid species. *Cerambyx welensii* Küster, 1845 (Coleoptera: Cerambycidae) showed variation in sex ratio and adult size under different ecological conditions [30], and twig-girdling beetles in the Onciderini tribe exhibited sex ratio shifts linked to seasonality and climate [31]. These findings support the hypothesis that low-temperature exposure may act as a cue affecting sex differentiation in CLB, warranting further study with larger cohorts.

Overall, comparative evidence for *A. chinensis* highlights the dual challenges of artificial rearing: elevated early larval mortality and reduced adult fitness on artificial diets, along with complex diapause break requirements that necessitate carefully calibrated chilling regimes. While much remains to be done to improve rearing performance, these findings refine our understanding of CLB biology and provide practical guidance for establishing laboratory colonies. Our results underscore the importance of integrating diet optimization with precise thermal management and appropriate chilling duration into rearing protocols of this species.

## Figures and Tables

**Table 1:** Location and number of *Anoplophora chinensis* adults collected from the wild during summer in 2023 and 2024.

Year	No. of Insects Collected	Location	Coordinates
2023	11 ♀ + 17 ♂	Jeollanam-do, Republic of Korea	34.98° N, 127.49° E
2024	15 ♀ + 10 ♂		
Total	26 ♀ + 27 ♂		

**Table 3:** Developmental duration of *Anoplophora chinensis* larval and pupal stages reared on artificial diet.

Chill Period			No. of Larvae Chilled	Percent (%)		Sex Ratio (F:M)	Duration (Weeks)		
Order	Duration (Weeks)	Temp. (°C)		Pupation	Emergence		Larva	Pupa	Total (Larva-Adult)
1st	12	10	39	7.7	100	1:0	32.67 ± 0.17	1.76 ± 0.05	34.43 ± 0.22
2nd	12	10	31	12.9	100	1:0	53.68 ± 0.30	2.25 ± 0.12	55.93 ± 0.36
3rd	16	10	15	60.0	100	1:0.8	85.24 ± 0.61	3.41 ± 0.16	88.65 ± 0.60

**Table 4:** Adult mean body weight (g) of *Anoplophora chinensis* reared on artificial diet and collected from the wild.

Sex	Artificial Diet		Wild Population		*p*-Value
	n	Weight (g)	n	Weight (g)	
Female	30	1.06 ± 0.02	15	1.28 ± 0.08 **	0.001
Male	18	0.67 ± 0.02	10	0.79 ± 0.06 *	0.036

Within the same row, * and ** indicate significant difference at the 95% and 99% level of confidence, respectively.

**Table 5:** Effects of 10 °C chilling for different durations (weeks) on pupation, adult emergence, and mortality in *Anoplophora chinensis* larvae.

Chilling Duration (Weeks)	No. of Larvae Chilled	Percent (%)			Sex Ratio (F:M)
		Mortality	Pupation	Emergence	
9	15	6.7 a	0.0 a	-	-
12	18	5.6 a	16.7 ab	100.0	1:0.5
14	20	10.0 a	45.0 bc	100.0	1:0.5
16	20	5.0 a	55.0 cd	100.0	1:0.83
19	18	16.7 a	44.4 bc	100.0	1:1
Non-chill	17	17.6 a	5.9 a	0.0	-
Statistical values		*χ*^2^ = 2.785 *df* = 5 *p* = 0.73	*χ*^2^ = 22.699 *df* = 5 *p* < 0.01		

Within the same column, different letters indicate significant differences at the 95% and 99% level of confidence.

**Table 6:** Effect of chilling temperature on pupation, adult emergence, and mortality in *Anoplophora chinensis* larvae.

Year	Chilling Temperature (°C)	No. of Larvae Chilled	Percent (%)			Sex Ratio (F:M)
			Mortality	Pupation	Emergence	
2023 * (3rd chill)	5	11	0.0 a	0.0 a	-	-
	10	15	6.7 a	60.0 b	100.0	1:0.8
	Statistical values		*χ*^2^ ≈ 0 *df* = 1 *p* ≈ 1	*χ*^2^ = 9.207 *df* = 1 *p* = 0.002		
2024 (1st chill)	5	20	15.0 a	0.0 a	-	-
	10	20	5.0 a	55.0 b	100.0	1:0.83
	Statistical values		*χ*^2^ = 0.27 *df* = 1 *p* = 0.605	*χ*^2^ = 14.43 *df* = 1 *p* < 0.001		

* The larvae in the 2023 experiment had undergone two prior chilling periods; Different letters indicate a significant difference at 95% level of confidence. Chilling duration was 16 weeks for both experiment years.

## Data Availability

The original contributions presented in this study are included in the article. Further inquiries can be directed to the corresponding author.

## Appendix A

**Table A1:** Supplement Facts: Ingredients in one vitamin mix tablet (NOW Foods, Daily Vits™, Multi Vitamin & Mineral), as provided by the supplier.

Ingredients	Amount per Capsule
Vitamin A (95% as Retinyl Palmitate and 5% as Beta-Carotene)	1500 mcg
Vitamin C (as Ascorbic Acid)	60 mg
Vitamin D (as Ergocalciferol)	100 mcg (400 IU)
Vitamin E (as d-alpha Tocopheryl Succinate)	20 mg
Vitamin K (as Phytonadione K-1)	80 mcg
Thiamin (Vitamin B-1) (from Thiamin HCl)	1.5 mg
Riboflavin (Vitamin B-2)	1.7 mg
Niacin (Vitamin B-3) (as Niacinamide)	20 mg
Vitamin B-6 (from Pyridoxine HCl)	2 mg
Folate	680 mcg DFE (400 mcg folic acid)
Vitamin B-12 (as Methylcobalamin)	18 mcg
Biotin	300 mcg
Pantothenic Acid (Vitamin B-5) (from Calcium Pantothenate)	10 mg
Calcium [from Calcium Carbonate (Aquamin^®^ Seaweed Derived Minerals)]	20 mg
Iodine (from Potassium Iodide)	150 mcg
Magnesium (from Magnesium Oxide and Aquamin^®^ Seaweed Derived Minerals)	15 mg
Zinc (from Zinc Bisglycinate) (TRAACS™)	10 mg
Selenium (from L-Selenomethionine)	35 mcg
Copper (from Copper Bisglycinate) (TRAACS™)	1 mg
Manganese (from Manganese Bisglycinate) (TRAACS™)	2 mg
Chromium (from Chromium Picolinate)	120 mcg
Molybdenum (from Sodium Molybdate)	75 mcg
Potassium (from Potassium Chloride)	10 mg
Organic Fruit & Veggie Blend (Organic Cauliflower, Organic Spinach, Organic Carrot, Organic Beet, Organic Tomato, Organic Blueberry, Organic Cranberry, Organic Cherry, Organic Raspberry and Organic Strawberry)	50 mg
Lutein (from Marigold Flowers Extract) (*Tagetes erecta*) (FloraGLO^®^)	100 mcg
Lycopene (from Tomato Extract)	100 mcg
